# Effect of graphene sheets on the physico-chemical properties of nanocrystallite ceria

**DOI:** 10.1098/rsos.241771

**Published:** 2025-03-12

**Authors:** Elena A. Trusova, Ivan Ponomarev, Evgeny Shelekhov

**Affiliations:** ^1^ Laboratory of Functional Ceramics, FSBSI Baikov Institute of Metallurgy and Materials Science of the Russian Academy of Sciences, Moskva, Russia; ^2^ National University of Science and Technology, MISIS, Moscow, Russia

**Keywords:** graphene–ceria composite, oxygen-free graphene, nano-ceria, dilatometry of nanopowders, sol-gel

## Abstract

Currently, replacing expensive and short-lived materials for supercapacitors based on RuO_2_ with more cost-effective and high-performance materials that remain operational after a large number of cycles is a challenge. Cerium-based materials are the most attractive alternative because of cerium’s ability to quickly change oxidation state. This work proposes the synthesis of nanostructured graphene–ceria composite and studies its morphological features arising under the impact of oxygen-free graphene. The mechanism of formation of nano-ceria crystallites when the sol–gel transition occurs on the surface of graphene sheets is also considered. It has been proven that the introduction of 0.5–0.6 wt% graphene sheets into nano-ceria ensures the preservation of its single-phase state, while simultaneously increasing its dispersion. Using the dilatometry method, it has been determined that uniformly distributed sheets of oxygen-free graphene lead to a decrease in temperature of the beginning of composite sintering by 175°C compared with pure nano-ceria and increase the shrinkage value by two times, which, in turn, should promote better sintering. This study fills a gap in the synthesis and characterization of oxygen-free graphene composites, promising raw materials for small devices and large power plants.

## 1. Introduction

Currently, the energy industry lacks materials for high-efficiency supercapacitors with increased charge capacity, which is needed in the design of energy storage devices for today’s small electronics [1,2]. Almost all modern supercapacitors suffer from a number of disadvantages, the main one being the low charge density compared with conventional batteries. Recently, it has been shown that the use of graphene as a component of materials for electrodes of electrochemical supercapacitors allows achieving high device capacity and excellent stability in operation (up to 2000 cycles; [3–5]). Graphene is one of the ideal electrode materials for supercapacitors due to its high specific surface area and electrical conductivity, porosity and excellent chemical stability, non-toxicity, controllability and versatility, making them promising candidates for energy storage applications [6,7]. Currently, the most commonly used method for obtaining graphene is the reduction of graphene oxide, despite the fact that this process is multi-stage, difficult and expensive [8,9]. However, as is known, the strong π–π interaction between graphene sheets during reduction makes graphene prone to agglomeration, which significantly reduces its capacity. Therefore, preventing the aggregation of graphene sheets is one of the urgent problems, the solution of which will bring us closer to the creation of materials with increased capacitance for new supercapacitors.

To prevent aggregation and enhance the search for acceptable nanocarbon species, Lu *et al*. [10] proposed to solve the problem of ultra-low specific capacitance and low performance caused by the close packing of graphene nanosheets and the tortuous ion diffusion path in graphene-based electrodes. For this purpose, a three-dimensional hierarchically porous graphene fibre was developed with the inclusion of perforated graphene for more efficient use of its surface. The incorporation of metal oxide particles into the space between graphene sheets has been proposed [11,12]. However, in this case, a modified Hummers method, the only method currently used on an industrial scale, was used to obtain the graphene species. Unfortunately, it uses highly toxic hydrazine, aggressive KMnO_4_ and sulfuric acid, as well as requiring high water consumption. In this case, the result is oxidized or reduced-oxidized graphene, in which the *sp*^2^-electron system is irreversibly damaged. The introduction of this graphene species into a composite improves the properties of the material only by increasing the dispersion of the metal-oxide component but practically does not contribute to the electronic structure of the hybrid.

An important component of lithium–air batteries is the oxygen electrode, which ensures the reduction of oxygen during the discharge process and the oxidation of Li_2_O_2_ during the charging process, which largely depends on the mobility of electrons in the material [13]. To ensure effective operation, the graphene in the composite must contain a minimum amount of oxygen and be evenly distributed throughout the volume of the material; however, its content should not exceed 2 wt%. Compliance with these two requirements presents a number of difficulties. As mentioned above, the well-layered structure of graphene and the mesoporous structure of the metal oxide component of the material will contribute to meeting these requirements. As a result, an increase in catalytic activity and charge capacity can be expected.

For example, due to the layered nanostructure of a hybrid material based on graphene and MnO_2_, high rates of transfer of electrolyte ions and especially electrons through the electrode matrix were achieved, which made it possible to create a material with excellent electrochemical characteristics. The capacity of such an electrode remained at 95% of the initial value even after 15 000 cycles [11]. The structures in which nanometre layers of graphene and nickel, cobalt or ruthenium oxides alternate also attract the attention of developers as promising, and success can be expected in this area in the near future. All of the listed composites based on graphene and metal oxides are currently assessed as promising for the development of materials based on them with improved performance properties for microelectronics, photovoltaic systems, chemical sensors, energy storage and conversion devices.

The typical structure of a supercapacitor mainly includes current collector, positive and negative electrodes, electrolyte and separator, in which the electrodes are the key components that affect the performance of the supercapacitor. They should have a large specific surface area and high electron conductivity, which can ensure efficient penetration of electrolyte ions and fast electron transfer. Therefore, the search for ways to use nanostructured composites based on graphene and ceria for the production of supercapacitors and lithium-ion battery electrodes continues. In addition to the listed areas of research on the use of such hybrid structures, a tendency has emerged to use the capabilities of their unique structure and electronic properties in gas sensors, drug carriers, (photo)catalysts, nanocoatings for corrosion protection, etc. [13–17]. Moreover, in the latter cases, the increased rate of electronic exchange processes in hybrid structures involving oxygen-free graphene becomes of great importance; only this graphene species is capable of imparting this interesting feature to composites.

Another component of the hybrid structure, nanostructured ceria is known as a photocatalyst due to its excellent physical and chemical properties such as non-toxicity, chemical stability, low cost, high oxidizing capacity and high electron transfer capacity [18]. As a rule, the unique properties of nanosized ceria are directly related to its size and differ sharply from its bulk counterparts. Highly dispersed ceria nanoparticles of small size are ideal for catalytic applications [19]. However, the high surface energy due to the nanosized ceria particles will cause a strong tendency to aggregate, which significantly reduces their surface area and leads to a marked decrease in their catalytic activity [20]. Therefore, in order to increase the stability and reusability of ceria nanoparticles, suitable support is required to secure and stabilize them in order to avoid the above-mentioned problems during use [21].

As an alternative to traditional substrates, researchers began to consider graphene–CeO_2_ composite as a material with a unique two-dimensional structure, large surface area and excellent electrical conductivity, high chemical and thermal stability, and excellent adsorption capacity [22,23]. The synergistic effect was identified; it consisted of the fact that when metal oxide nanoparticles are fixed on graphene, the intensity of charge transfers across the interface between these two components increases. This leads to a significant increase in catalytic activity and even to the appearance of new properties that none of the components individually has [24–26]. Thus, the problem arises of creating a new architecture that contributes to the manifestation of the best properties of both components in the new material.

A number of works in recent years report success in the creation of materials based on ceria and various graphene species for a wide range of applications. Thus, in the work [27], it was shown that the inclusion of graphene in ceria increases the thermal stability of the electrode while almost completely (up to 98%) maintaining the efficiency of the supercapacitor. A material for an electrochemical sensor based on CeO_2_ and reduced oxidized graphene was proposed for the determination of tryptophan in food products and biological objects [28].

In search of ways to improve the electronic properties, to increase the thermal, optical and photocatalytic characteristics, ceria nanoparticles were doped with Fe^3+^, Zr^4+^ and Zn^2+^ ions (4.0−7.5 atomic %), isomorphically embedded in its lattice by the hydrothermal method or standard mechanical milling [29–31]. It was found that the presence of Fe^3+^, Zr^4+^ and Zn^2+^ atoms in the matrix leads to a change in the band gap width of ceria and an increase in its photocatalytic activity.

In the work [32], a nanostructured additive based on oxidized graphene and nano-CeO_2_ was proposed to protect epoxy coatings from atmospheric exposure. All of these works use oxidized graphene or reduced oxidized graphene, and they are united by the Hummers method (or modified Hummers method), which uses such aggressive reagents as KMnO_4_, hydrochloric and sulfuric acids, concentrated hydrogen peroxide and highly toxic hydrazine. In addition to the high toxicity of the reagents, explosion hazard and high water consumption, this method requires large costs for labour protection, environmental protection and social security of workers. In no case are the ways of removing K and Cl (using cerium chloride) discussed. However, practice shows that reduced oxidized graphene does not have the full range of unique electronic properties inherent in oxygen-free graphene. In addition, the synthesis of oxidized graphene and its subsequent reduction requires severe conditions—strong oxidizing agents such as KMnO_4_ and sulfuric acid, as well as toxic reducing agents.

In the work [33], it was shown that it is very important to develop appropriate technological modes of compaction and sintering that make it possible to preserve the electrical properties of the composite when producing electrode material from a powder composite. The sintering of graphene–Me_*x*_O_*y*_ materials has some limitations related to the thermal stability of graphene. To overcome them, researchers are looking for new sintering methods to lower the temperature and reduce the duration of the process. Such methods include hot pressing, spark plasma sintering and microwave sintering [34].

In the work [35], the ceria nanocrystals were grown on the graphene sheets, and it was shown that the resulting hybrid structures exhibit unique photocatalytic activity. Spherical ceria nanocrystals with dimensions of 10−12 nm had defects on their surface in the form of oxygen vacancies that appeared as a result of the interaction of nano-ceria with graphene, which led to a decrease in the width of the ceria band gap. It was found that the combination of defects in ceria nanocrystals with the optimal number of graphene sheets had a significant effect on the properties of the resulting hybrid structures, such as improved optical, photocatalytic and photocapacitive characteristics.

Despite a number of attempts to obtain graphene–сeria composites and determine some of their electrical properties, there are no general views on the nature of the interaction of graphene, especially oxygen-free, in a hybrid structure. In each individual case, a statement of facts is given that does not allow one to form an idea of the nature of the interactions and the origin of the effects. The fact that the Hummers method is the only one used industrially has long called for an extensive search for alternatives that are more environmentally friendly and cost-effective. In addition, due to the fact that most of the known publications are devoted to oxidized or reduced oxidized graphene, which *a priori* does not have the electron properties of oxygen-free graphene, the formulation of this work is timely, and its results can become the basis for an environmentally friendly and cost-effective technology for the production of graphene–ceramic materials for a wide range of purposes.

Interesting results were presented in the work [36], where a hydrothermal method for producing a nanocomposite based on reduced oxidized graphene and ceria is reported. It was found that the resulting composite has a narrower band gap compared with pure nano-ceria and provides faster electron transport. As a result of the improved electronic properties of the composite, it exhibited increased photocatalytic activity in the decolorization of Rhodamine B under the influence of sunlight. The developed nanocomposite also exhibited improved capacitive properties compared with pure ceria nanoparticles, apparently due to the presence of graphene sheets.

Previously, we proposed a method for obtaining hybrid nanostructures based on oxygen-free (non-oxidized) graphene and nanocrystalline metal oxides [37], which combines sol–gel synthesis and ultrasonic exfoliation of graphene sheets from the surface of synthetic graphite in organic or organic–inorganic media [38–42]. The purpose of this study is to develop a method for the synthesis of nanostructured graphene–ceria hybrid powder under mild conditions, study its physico-chemical properties, determine the role of graphene and its content in the formation of the hybrid structure, as well as to compare the sintering behaviour of nanostructured ceria powders and a composite based on it with oxygen-free graphene. The initial powders of the graphene–ceria composite were obtained by a method that combined sol–gel synthesis and ultrasonic exfoliation of the graphene sheets from the surface of synthetic graphite in an N,N-dimethyloctylamine-aqua (DMOA-aqua) emulsion [37]. Subsequently, these powders were investigated using the dilatometric method to elucidate the role of graphene in the dynamics of sintering and determining the most optimal temperature mode. The results obtained will be useful not only for developers of new composites but also for those researchers who have already obtained practical results but did not focus on the nature and origin of the effects, as well as their mechanisms and driving forces.

## 2. Experimental

### 2.1. Sonochemical preparation of graphene suspension in dimethyloctylamine-aqua emulsion

An oxygen-free graphene suspension was obtained by a sonochemical method developed earlier [39]. Synthetic graphite (NPO Unichimtek, Russia) with particles 600−800 µm in size was used as a carbon source, and the residual content of sulfur and chlorine was 10 ppm. An emulsion of DMOA (Acros Organics, CAS 7378-99-6, purity is 95%) in aqua (0.005−0.009 M) was used as the dispersion medium, which was acidified with nitric acid (Prime Chemicals Group, Russia, GOST 11125-84, purity is 70%) to pH value equal to 3. The resulting suspension of graphite in an aqua-organic medium was treated in an ultrasonic bath Sonoswiss SW1H with a power of 200 W for 60 min at a temperature of 60°C. The resulting mixed suspension of graphene–graphite was separated by stepwise decantation: after 2 and 22−24 h sedimentation, thus obtaining a light fraction of graphene, which was used for further synthesis. The degree of graphite conversion in all experiments was 3%; unreacted graphite after decantation and drying was returned for reuse. The influence of the duration of graphite irradiation on the graphene content in a dispersion medium was studied by repeatedly applying it to a sample of commercial aluminium oxide as a standard carrier and determining the weight gain.

### 2.2. Synthesis of the graphene–ceria composite

For the synthesis of graphene–ceria composites, a method based on a combination of sol–gel synthesis of metal oxide nanostructures and ultrasonic exfoliation of graphene sheets in an organic-aqua medium was used [38]. A sol was synthesized using cerium (III) nitrate hexahydrate (Ce(NO_3_)_3_∙6H_2_O, LANHIT, Russia, CAS: 10294-41-4, purity is 99.9%) as a metal source. Its aqueous 0.05 M solution, heated to 80−90°C and vigorous stirring, was mixed with an alcohol solution of the sol stabilizer, which was DMOA (Acros Organics, CAS 7378-99-6, purity is 95%), at a molar ratio of DMOA : Ce = 1. A complexing agent, acetylacetone (AcAcH, ChimMed, Russia, GOST 10259-78, purity is 99.5%), was introduced into the resulting solution at a molar ratio of AcAcH : Ce = 2.4 also with stirring and heating (80–90°C). The sol thus obtained was subsequently used to obtain сeria nanopowder and graphene–CeO_2_ composite. In the latter case, the resulting sol was mixed with a decanted suspension of oxygen-free graphene during stirring and heating. The resulting colloid was evaporated with stirring and heating to 90−95°C to form a gel, which in one case was heat treated in air at 350°C for 30 min, and in the other one at 500°C for 1 h on air. In both cases, the light yellow fine powders were obtained.

### 2.3. Characterization of obtained colloids and powders

The morphology of the synthesized graphene powders and suspensions was studied by transmission electron microscopy (TEM) using a LEO 912 ab Omega Carl Zeiss instrument, which also provided selected area electron diffraction (SAED) for all samples. A detailed study of the structure of graphene sheets and composite powders was carried out using high-resolution TEM (HRTEM) on a JEM 2010 instrument (JEOL Ltd) with attachments for energy dispersive spectrometry (EDS, Inca, Oxford Instruments) and characteristic electron energy loss spectroscopy (EELS, GIF Quantum, Gatan Inc.) and also JEOL JEM-2200FS instrument.

X-ray diffraction (XRD) studies were carried out on a DRON-3 diffractometer using the Bragg–Brentano geometry. Bragg–Brentano (symmetric reflection geometry, 2*θ*/*θ* scan), i.e. the angle of incidence of radiation on the surface of the sample is always equal to the angle of exit (mirror, also known as focusing scheme); the rotation speed of the sample is half the speed of the detector. When studying nanopowders, in the diffraction of which the reflections should be broadened *a priori*, it is sufficient to use a shooting step of 0.1°. Cu radiation with a wavelength of 1.54178 Å was used, and the shooting interval was 10−125°. The goniometer rotation speed was 16° s^−1^. Other shooting parameters: the number of shooting points was 1151 and the exposure of one point was 3 s. The crystallite size and the fraction of micro strains were calculated by the Scherer method. The Joint Committee on Powder Diffraction Standards (JCPDS) card files were used for phase identification. The carbon content of the composite samples was determined using a Leco model CS-600 instrument.

### 2.4. Dilatometric study of the synthesized nanostructured powders

The dilatometry study of the synthesized nanostructured powders was carried out using a DIL 402 C Netzsch dilatometer (Netzsch, Germany) according to a previously developed method [42]. The obtained, from nanopowders, cylindrical green bodies, with a diameter of 5 mm and a height of 2.5 mm, were used for the study. The thermocouple (tungsten–rhenium alloy) was located near the sample, and its temperature was accurately recorded; the second thermocouple (tungsten–rhenium alloy) was in the chamber with the heater. This chamber is separated from the working chamber and filled with argon. The argon flow through the furnaces was 70 ml min^−1^, heating was carried out up to 1650°C at a rate of 5, 10 and 20°C min^−1^ after which cooling was carried out at a rate of 20°C min^−1^.

## 3. Results and discussion

**Figure 1.**
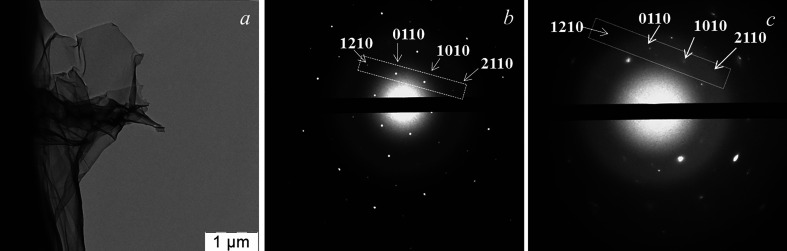
TEM data for a graphene suspension in DMOA-aqua: (*a*) bright-field image of the sheet and (*b,c*) SAED on the different regions of a sheet, shown in (*a*), the characteristic reflexes are marked with the frames.

**Figure 2.**
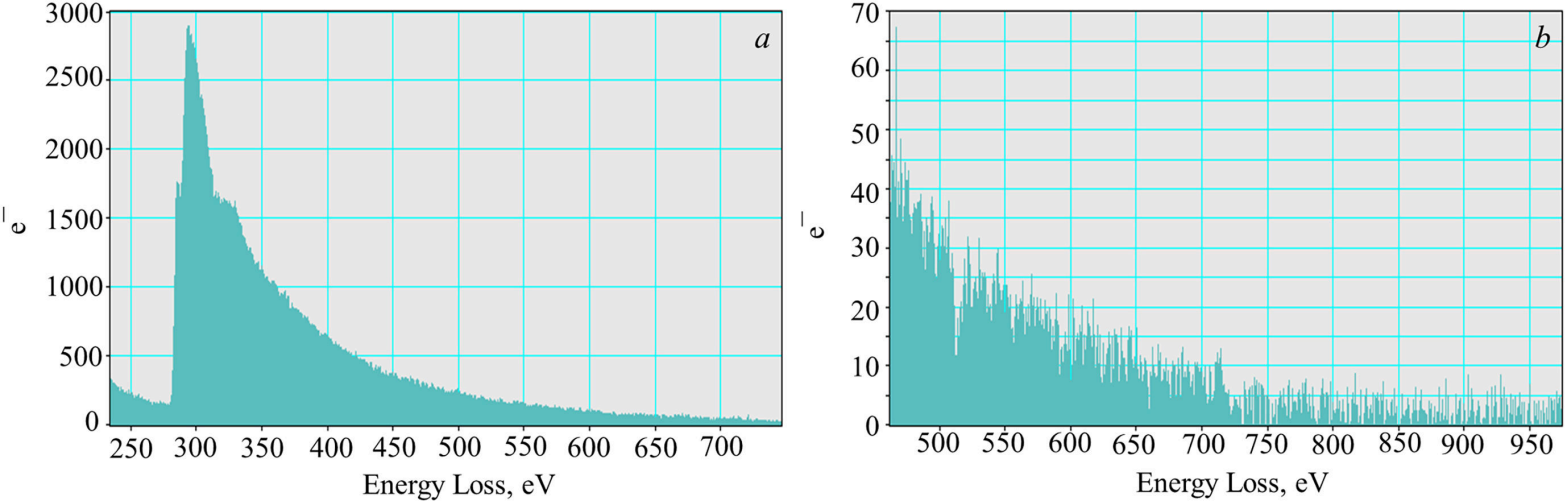
EELS analysis of the suspension (*a*) and enlarged region above energy loss value 450 eV (*b*).

The TEM image in figure 1*a* shows that the graphene suspension consisted of translucent deformed sheets with micrometre linear dimensions, which had randomly oriented folds. This configuration allows the sheets to maintain a minimum surface energy and maximum thermodynamic stability. As shown by SAED in figure 1*b*,*c*, the sheets had a variable thickness and consisted of the regions of multi-layer and monolayer graphene spaces. The last assertion can be made because of the intensity ratios of the characteristic reflections: *I*_0110_/*I*_1210_ > 1 and *I*_1010_/*I*_2110_ > 1, which are characteristic of monolayer regions [43]. According to the EELS analysis shown in figure 2*a*, graphene sheets practically did not have oxygen-containing functional groups on their surface: there were no bands in the 532 eV region characteristic of oxidized graphene, which is clearly shown in the enlarged image of the region over 450 eV (figure 2*b*). At the same time, a broad peak centred at 284 eV testified to the 1*s* → π* transition and indicated the presence of *sp*^2^-carbon atoms in the system [44,45].

**Figure 3.**
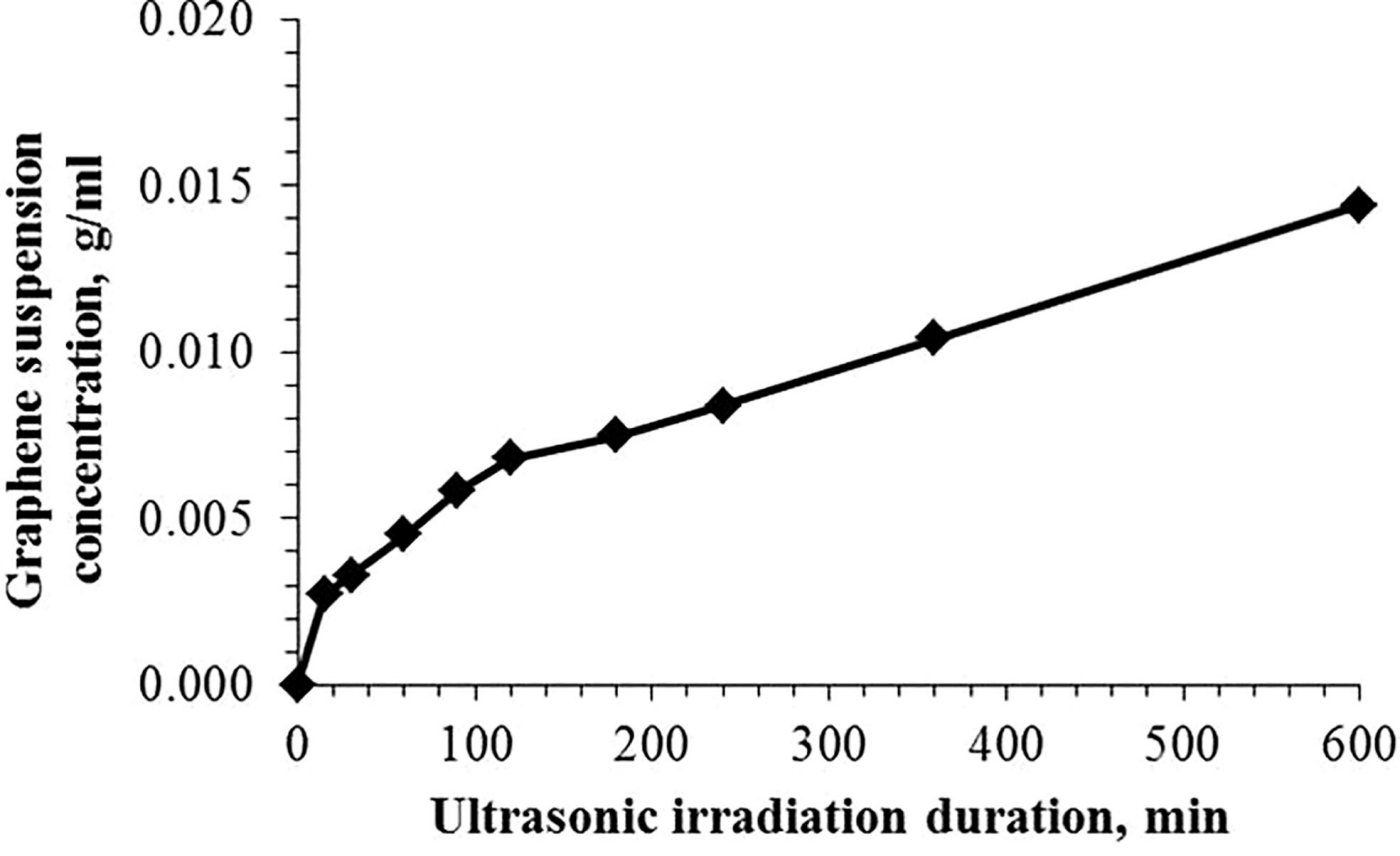
The impact of the duration of ultrasonic irradiation of graphite on the graphene concentration in suspension at pH value of 3.

The effect of the duration of ultrasonic irradiation on the graphene concentration in suspension was studied in the range of 15 to 600 min. Figure 3 shows that increase in the content of graphene sheets occurs nonlinearly; the curve has an inflection in the region of 100 min. With a further increase in the duration of treatment, the increase in concentration slows down, apparently due to the implementation of the reverse process of association of light sheets into larger particles, which we separate during decantation.

**Figure 4.**
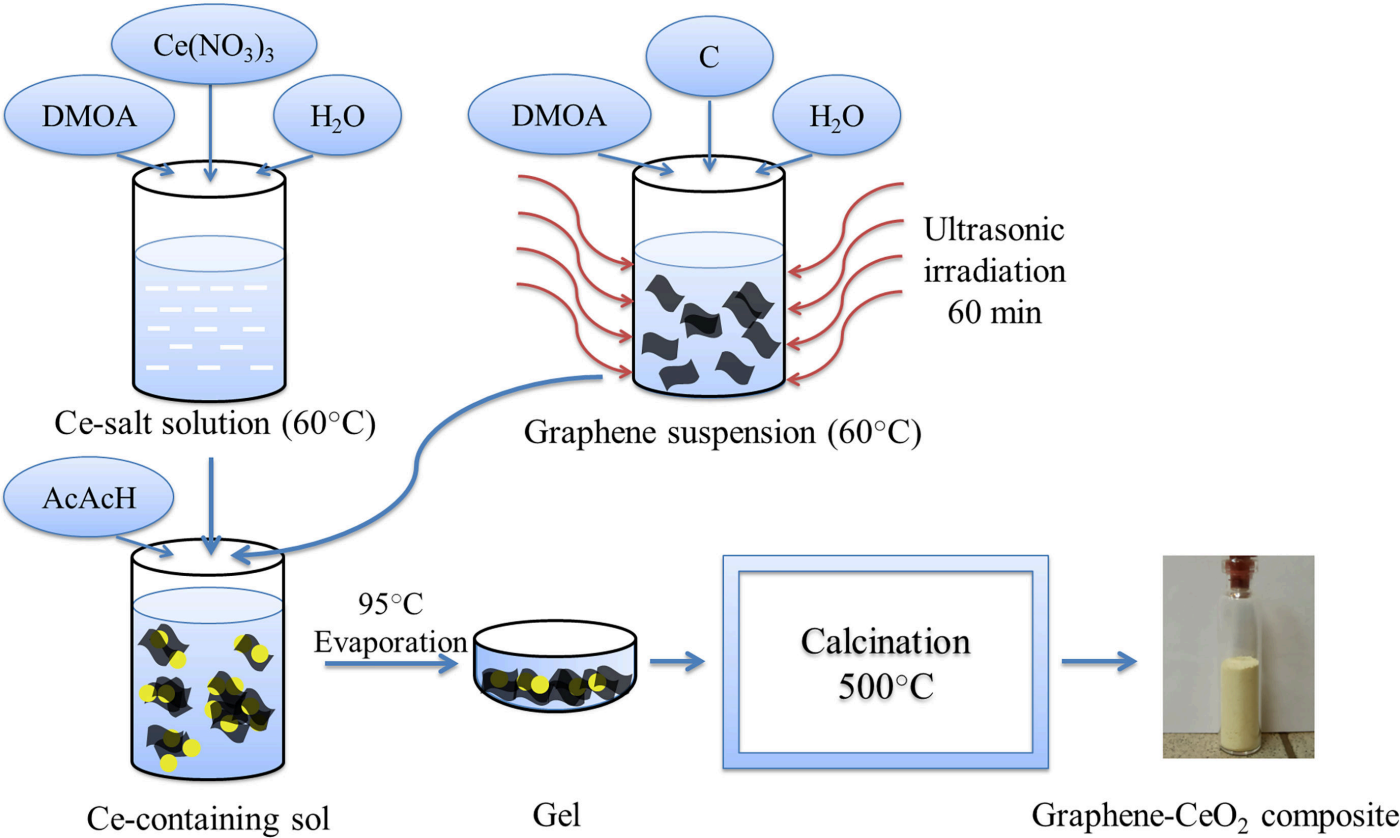
Scheme for the synthesis of a graphene–CeO_2_ composite from a Ce-containing sol and a graphene suspension in an organic-aqua medium (DMOA-aqua).

Figure 4 shows the scheme for the synthesis of graphene–ceria composites, described in the experimental part. According to the elemental analysis data, the content of graphene in the composite in the entire experiment was 0.5−0.6 wt%. The study of the morphology of xerogel-intermediate, the heat treatment of which was suspended when the temperature reached 350°C, was carried out to research the dynamics of the formation of a nanostructured composite during calcination and crystallization. At this temperature, the weight loss of the sample stopped, which indicated the complete decomposition of the organic–inorganic oligomeric gel and the formation of an amorphous intermediate xerogel.

**Figure 5.**
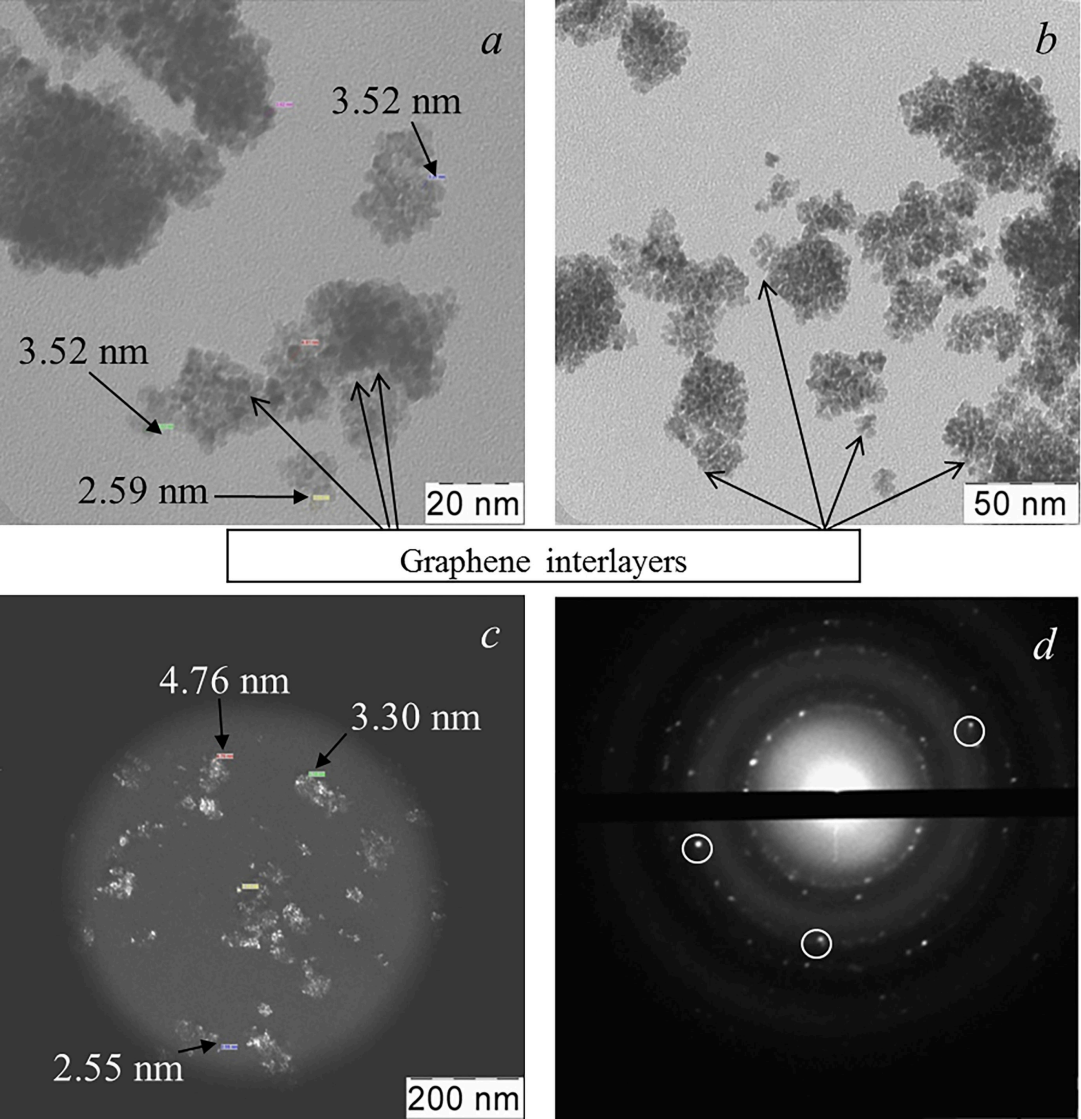
TEM image of a xerogel-intermediate (at 350°C), a precursor of the graphene–ceria composite: bright-field (*a,b*) and dark-field (*c*) images, and SAED of the sample area shown in *c (d*); the graphene reflections are highlighted by circles. Arrows in (*a*) and (*b*) indicate the automatically measured thickness of the graphene sheets on the crystallites in agglomerates of the xerogels-intermediate. The arrows in (*c*) indicate the automatically determined dimensions of individual crystallites in the agglomerate.

Figure 5 shows the TEM data for the resulting composite xerogel after the complete decomposition of the organic components of the reaction mixture during heat treatment at 350°C. Figure 5*a,b* clearly shows that the xerogel powder consisted of agglomerates with 40−70 nm in size, including discrete particles with 2−7 nm in dimension. In this case, inside the agglomerates, almost all particles had the same dimensions and were separated by semitransparent interlayers with a thickness of 1−2 nm. The dark-field image also indicates the presence of discrete particles with dimensions of 2−7 nm in the composition of agglomerates (figure 5*c*). Electron diffraction in figure 5*d* shows that the powder particles consist of many differently oriented graphene sheets (the reflections are highlighted by circles) and nanoparticles of the weakly crystallized ceria phase.

**Figure 6.**
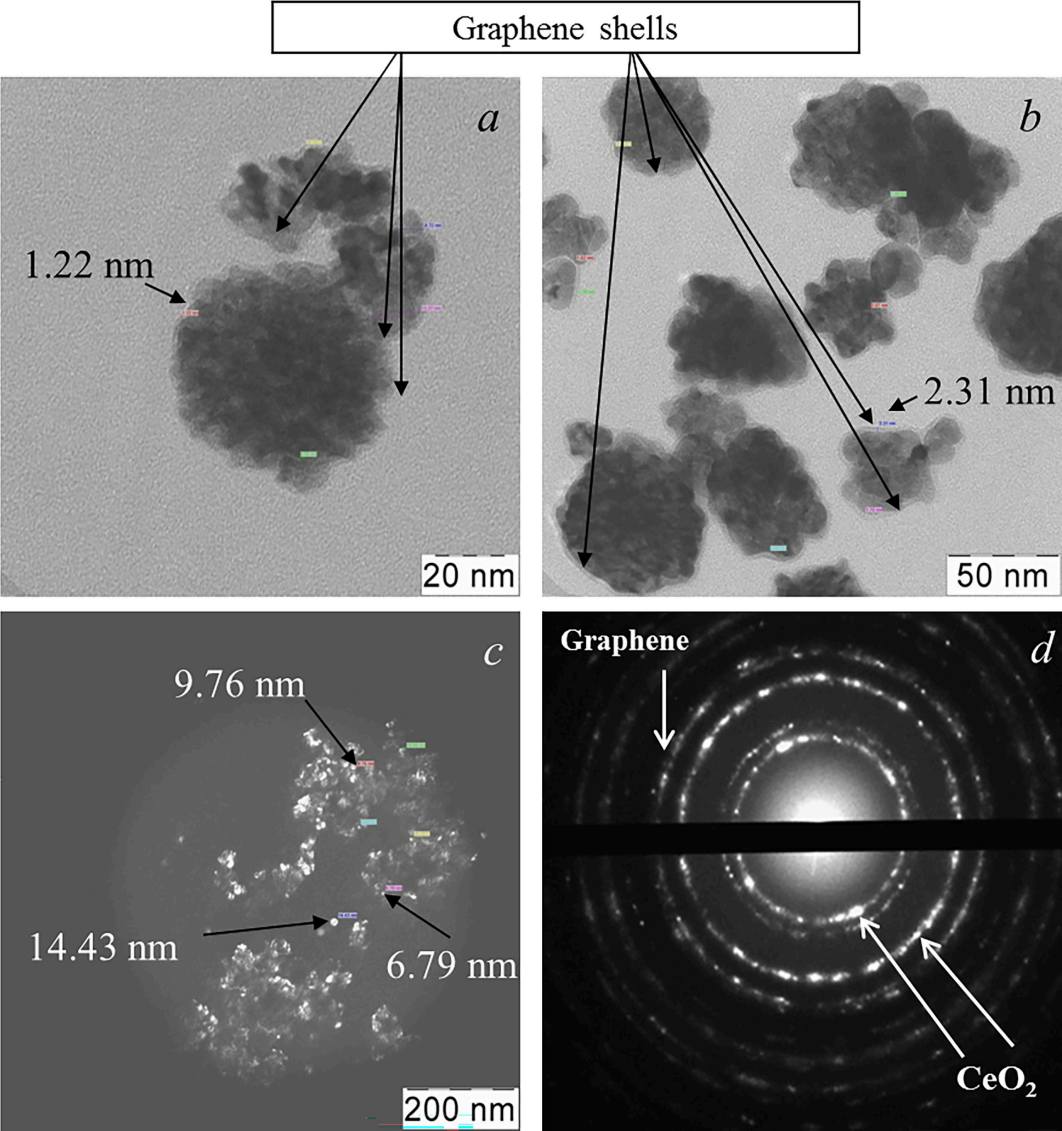
TEM data for the graphene–ceria composite calcined at 500°C: bright-field (the arrows indicate automatically measured sizes of ceria nanocrystals) (*a,b*), dark-field image (the arrows indicate the automatically determined sizes of individual ceria crystallites in the agglomerate) (*c*) and SAED (*d*). The content of graphene in the composite was 0.5 wt%.

The further heat treatment of composite powder at an increase in temperature to 500°C, at which it was kept for 1 h, led to the final crystallization of ceria in the presence of graphene and the formation of the composite architecture. The TEM data for crystallized composite obtained at 500°C, presented in figure 6, show that an increase in the heat treatment temperature does not lead to a significant coarsening of the graphene-containing agglomerates. However, the dimensions of crystallites inside these agglomerates increase up to 8−14 nm (figure 6*a*,*b*) which is also confirmed by the dark-field image in figure 6*c* with automatically measured crystallite dimensions (indicated by arrows). Figure 6*b* shows the thickness of graphene shells of agglomerates, which is 1−2 nm. SAED for the composite in figure 6*d* is the result of superposition of diffraction patterns for graphene and many differently oriented ceria crystallites.

**Figure 7.**
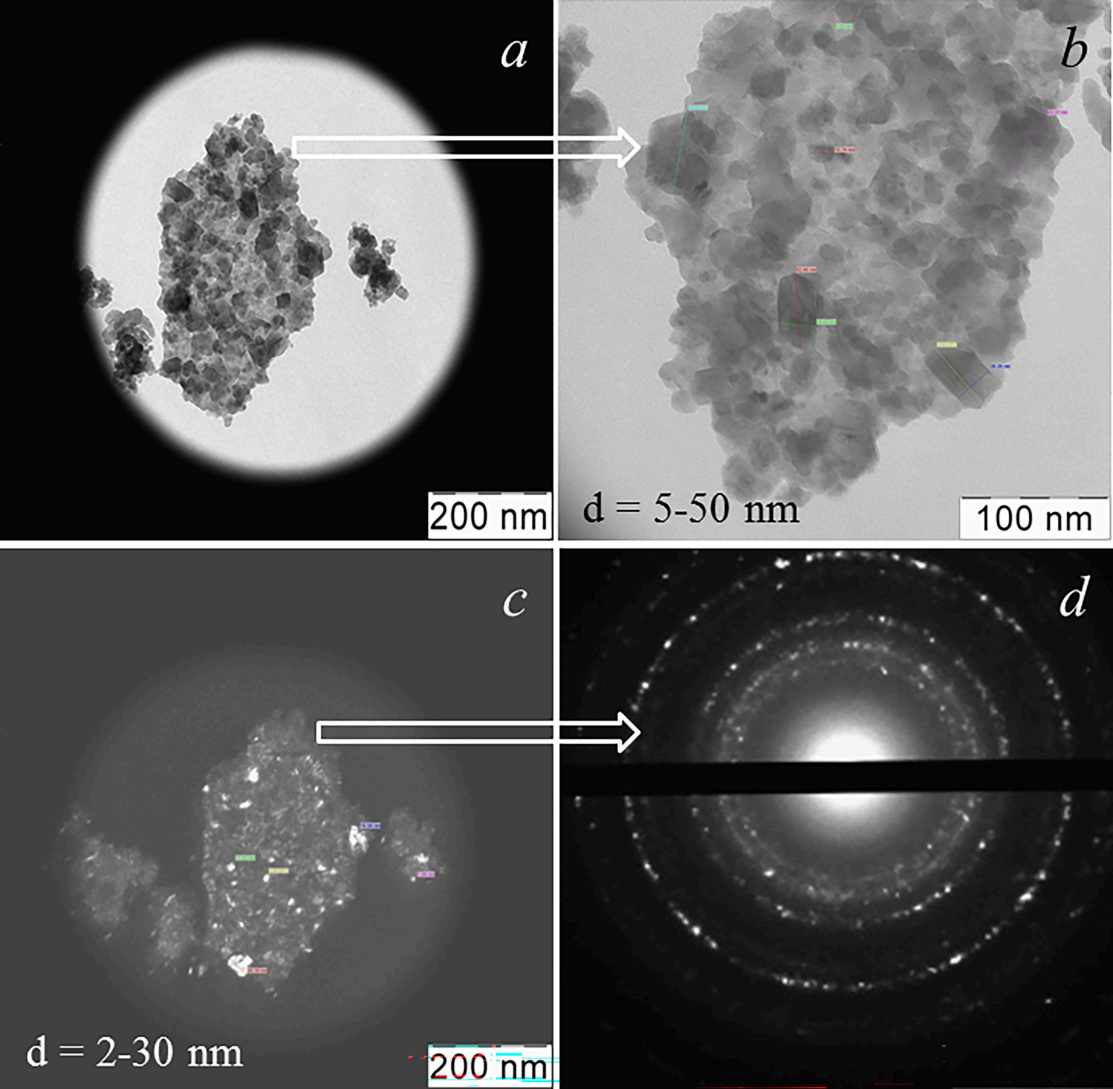
TEM data for pure nano-CeO_2_: bright-field (*a* and *b*) and dark-field (*c*) images and SAED for the sample area shown in *a* and *c* (*d*).

For comparison, figure 7 shows the TEM results for a pure ceria nano-powder obtained from the same sol as the composite. As can be clearly seen, the sample taken consisted of the agglomerates of nano- and sub-micrometre sizes (figure 7*a–c*) formed by differently oriented nanocrystals with dimensions of 2−50 nm. Obviously, in pure nano-ceria powder, the dimensions of both ceria crystallites and their agglomerates significantly (almost a multiple) exceed the same parameters for the composite. The electron diffraction analysis for the agglomerate shown in figure 7*c* allows us to conclude that ceria is well crystallized (figure 7*d*). However, the difference from the diffraction shown in figure 6*d* is also obvious: it consists of a higher ordering of crystals in the agglomerate and a higher intensity of reflections due, probably, to the larger sizes of ceria crystallites in a pure nanopowder.

**Figure 8.**
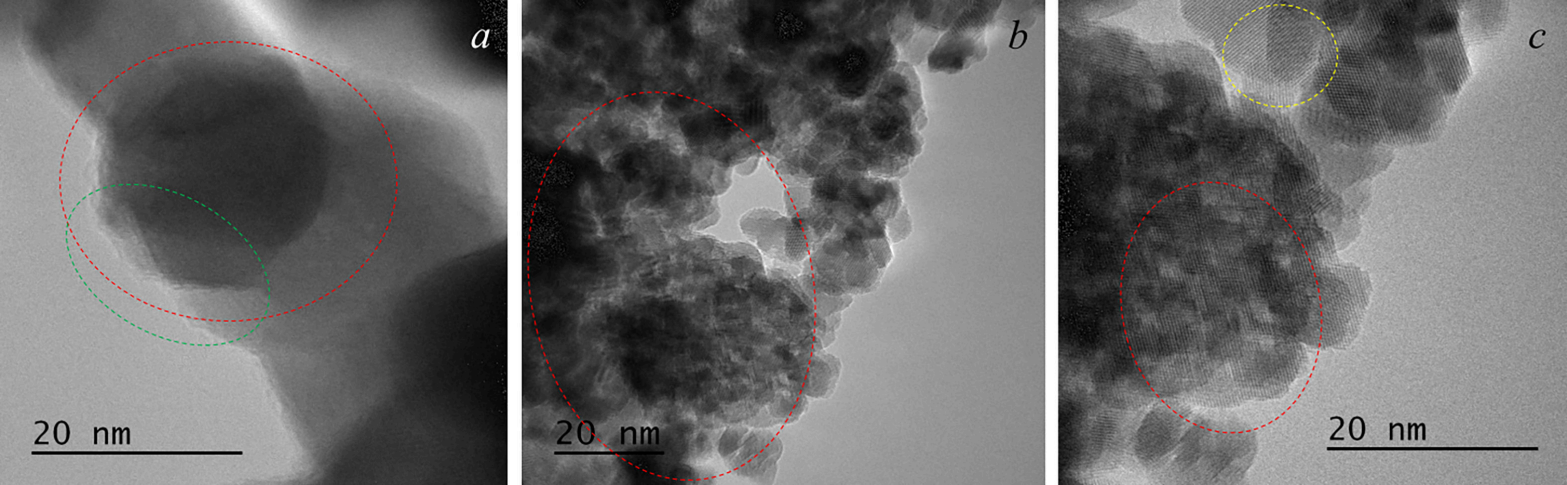
HRTEM images for graphene–ceria composite: the red ovals denote a characteristic moire on the surface of the nanoparticles, the green oval shows a semitransparent graphene sheet and the yellow oval highlights rounded crystallites. The images in (*a*)*–(c*) were obtained using the JEOL JEM 2010 instrument.

Figure 8 shows the results of a detailed HRTEM study of the graphene–ceria composite morphology. As can be seen, the composite powder consists of nanostructured agglomerates formed by two types of crystallites: regular cubic shape with dimensions of 10−20 nm (figure 8*a*) and rounded ones with dimensions of 8−10 nm (figure 8*b,c*), the latter predominating. The red ovals in figure 8*a–c* denote a characteristic moire on the surface of the nanoparticles, which indicates graphene sheets in the composite. In figure 8*c*, it is clearly seen that several rounded crystallites with sizes of 4−6 nm are grouped on a hexagonal semitransparent graphene sheet with a side of 12−15 nm (highlighted by a yellow oval). In figure 8*a*, it was possible to determine a semitransparent graphene sheet on the face of the ceria crystallite (highlighted by a green oval). Such morphology was not observed on pure ceria powder (figure 7) obtained from the same sol as the considered composite.

**Figure 9.**
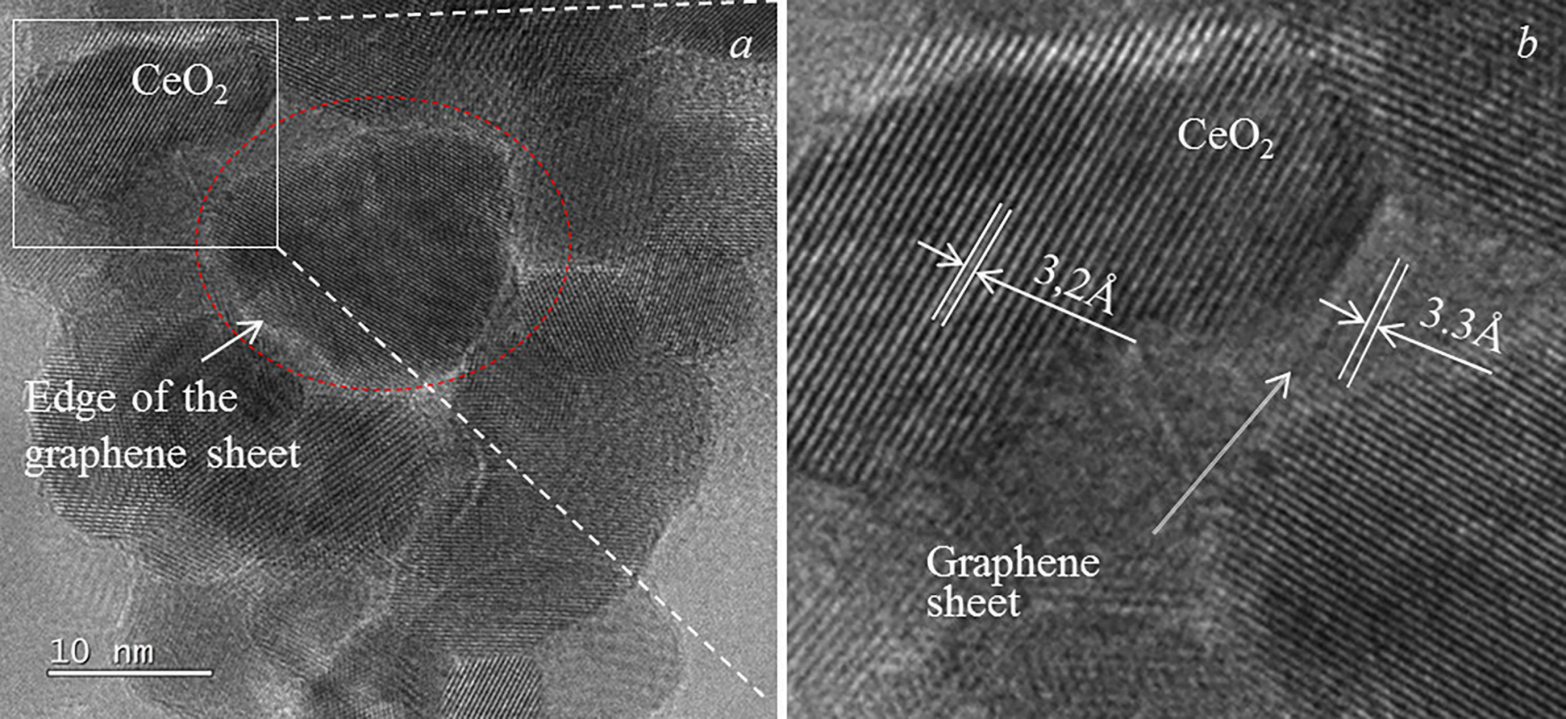
HRTEM image for graphene–ceria composite (*a*), and an enlarged fragment, highlighted in *a* by a square (*b*). The red oval shows an agglomerate of several ceria crystallites fixed on the bottom of graphene sheet. The image was obtained using the JEOL JEM-2200FS instrument.

It is interesting to consider a group of a few ceria crystallites in the centre of figure 9*a* under a graphene sheet (highlighted in red oval); the presence of the latter is indicated by moire (the edge of the sheet is marked with an arrow). Apparently, this group is an agglomerate with dimensions of approximately 12−13 nm with a common boundary along the contour, which in the picture looks like a lighter line. This image also clearly shows that the vast majority of сeria crystallites are discretely fixed on graphene sheets; this is evidenced by the light contours observed in almost every crystallite. Assessing the transparency of graphene sheets, we can say that their thickness is almost comparable with the parameters of the ceria crystal lattice and is one to two layers. An enlarged fragment of figure 9*a* is shown in figure 9*b*; it clearly demonstrates the arrangement of ceria crystallites and graphene sheets in the composite agglomerate, which has a multi-layer architecture. In the work [46], it was noted that such alternation of nanocomponents in the material contributes to the formation of increased electronic conductivity and acceleration of charge transfer reactions in lithium-ion batteries.

**Figure 10.**
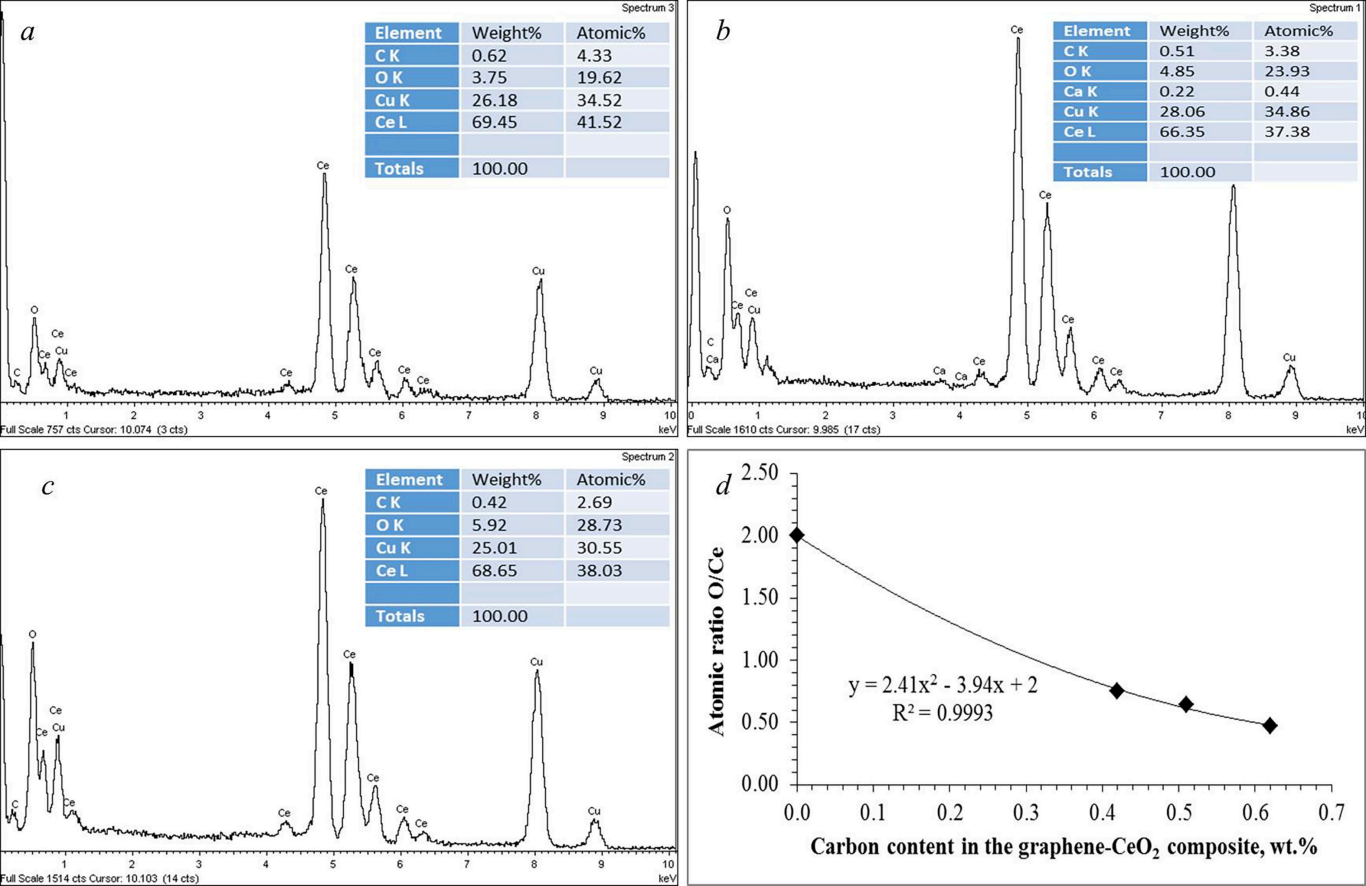
The results of an EDS analysis of a section of the composite sample shown in figure 8 (*a*–*c*). dependence of the O/Ce atomic ratio on the proportion of graphene introduced at the various local sites, according to the EDS analysis (*d*).

Determination of local effects from the graphene presence was carried out using EDS to study the area shown in figure 8*a* and its results are shown in figure 10*a*. The atomic ratio O/Ce in the area under consideration is 0.47, which contradicts the molecular formula CeO_2_. The carbon content in the area under consideration is 0.6 wt%, which is 1.5 times higher than the value obtained by the integral elemental analysis (0.4 wt%). The local carbon content values in other parts of the composite sample according to EDS analysis (figure 10*b*,*c*) were 0.51 ± 0.02 and 0.42 ± 0.02 wt%, respectively. This indicates that the ceria in the composite is non-stoichiometric. Apparently, some of the Ce^4+^-ions turn into the Ce^3+^-state, most likely during the decomposition of the organic–inorganic complex of the gel. However, it can be assumed that this phenomenon may be due to the crystallization of ceria on the graphene sheets, which leads to an increase in the number of oxygen vacancies in the crystal lattice, mainly on the crystallite surface.

This assumption is supported by the dependence of the O/Ce atomic ratio on the graphene content in the composite, which was determined from the EDS analysis data (table 1), shown in figure 10*d*. The dependence is nonlinear and is described by a polynomial of the second degree. In this case, the steepest slope of the curve corresponds to the initial section, which confirms the effect of the presence of graphene in the system. According to the dependence obtained, graphene directly affects the structure of ceria crystallites formed on it, namely, it promotes the formation of oxygen vacancies in crystallites, the number of which is proportional to the amount of graphene present in the contact area.

**Table 1.** Effect of the carbon content in the various sites of the graphene–ceria composite on the О/Се atomic ratio (according to EDS analysis data).

carbon content, wt%	0	0.42	0.51	0.62
atomic ratio O/Ce	2	0.75	0.64	0.47

**Figure 11.**
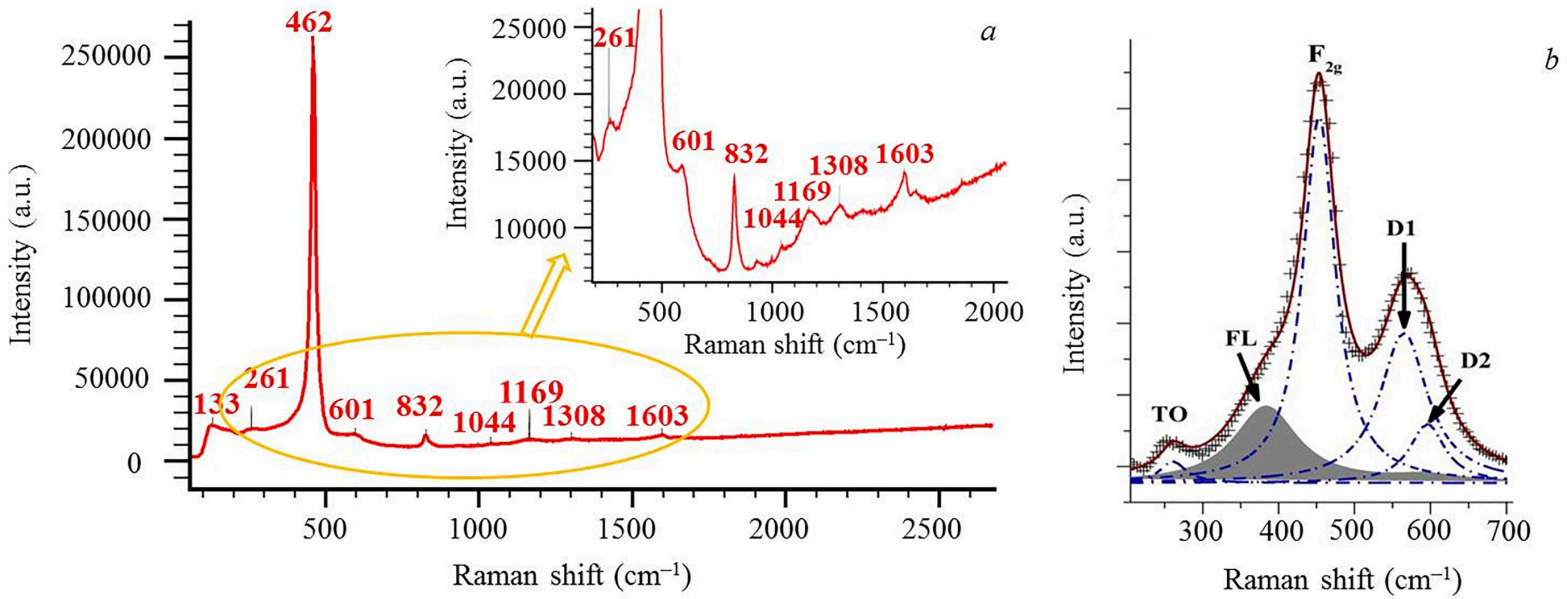
Raman spectrum of graphene–CeO_2_ composite nanopowder. The laser excitation line is 532 nm. General view (*a*) and decomposition of spectrum in the area of *ca* 300−700 cm^−1^ into three components (*b*). The area highlighted in yellow is shown in the inset.

Figure 11 shows the Raman spectrum of the graphene–ceria composite, where the line with a maximum in the region of 464 cm^−1^ dominates. It is a triple degenerate mode F_2g_ [47], which is associated with vibrations of four oxygen atoms around the Ce^4+^ cerium cation [48]. The 462 cm^−1^ line is sensitive to changes in the size of ceria particles: with a decrease in the crystallite size, this line broadens and its symmetry decreases [49]. Also in the spectrum, there are weaker bands with maxima in the regions of 260, 596, 1174 and 831 cm^−1^, which are not observed in the spectrum of single-crystal well-crystallized ceria. The bands with maxima in the regions of 260, 596 and 1174 cm^−1^ appear due to the combination of vibrational modes A_1g_, E_g_ and F_2g_ of the CeO_2_ lattice owing to defects.

The asymmetric band with a maximum in the region of 831 cm^−1^ refers to non-planar bridging peroxide complexes of oxygen adsorbed from air with two-electron defects [50]. A weak line with a maximum in the region of 260 cm^−1^ refers to a transverse optical (TO) phonon (figure 11*a*), which is forbidden in the Raman spectra of an ideal CeO_2_ crystal, but becomes active when defects in the crystal lattice occur, caused by the appearance of Ce^3+^ cations in the CeO_2_ lattice [51]. The wider weak band in the region of 570−610 cm^−1^ (in figure 11*b*, this band is marked as 596 cm^−1^) consists of two bands: D1 and D2 (figure 11*b*); a band with a maximum in the region of 542 cm^−1^ and relating to oxygen vacancies [52,53] and a band with a maximum in the region of 602 cm^−1^ related to the appearance of the Ce^3+^-cations in the ceria structure. Previously, it was determined that local disturbances in the lattice affect the size-dependent spectral characteristics of ceria nanocrystals [54]. In the region of 1170 cm^−1^, a broad band is observed, which is associated with the phonon repetition of a longitudinal optical phonon (2LO). The intensity of the D1 and 2LO bands with maxima at 596 and 1200 cm^–1^ increases with decreasing laser excitation length.

Thus, when excited by a laser with a wavelength of 785 nm, the intensity of the band located in the region of 570−610 cm^–1^ is less than 1% of the intensity of the band with a maximum in the region of 464 cm^–1^. At a laser radiation wavelength of 532 nm, the intensity is already approximately 5% of the intensity of the band with a maximum in the region of 464 cm^−1^. At a wavelength of 405 nm, the intensity of the band approaches 20% of the intensity of the band with a maximum in the region of 464 cm^−1^. The observed spectrum pattern is associated with the electron structure of oxygen vacancies in ceria: under resonant conditions, the D2 defect region is folded with the resonantly enhanced 1LO mode, and therefore, only the D1-related vacancy of the two defect bands can be resolved [55]. There are no bands in the spectrum due to graphene or its interaction with ceria, which are usually observed in the range 1200−1600 cm^−1^—D and G (graphene bands)—apparently due to the low content of graphene.

**Figure 12.**
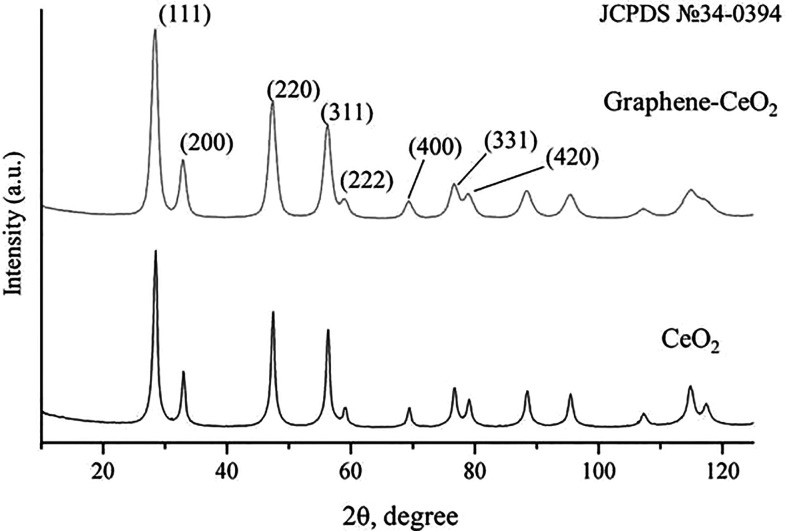
XRD patterns for pure nano-ceria and graphene–ceria composite powders. In both cases, identification was carried out using JCPDS card no. 34-0394.

XRD (figure 12) of both powders nano-ceria and graphene–ceria composite corresponded to the face-centred fluorite-type crystal lattice (JCPDS card no. 34-0394). The average crystallite size (according to Cauchy) of pure nano-ceria was 12.2 nm, in the composite this value was 9.0 nm. Differences were also observed in the proportion of deformations of the fluorite crystal lattice; in the composite, it was 30 times higher than in pure nano-ceria powder: 0.467 ± 0.003% and 0.016 ± 0.007%, respectively.

**Figure 13.**
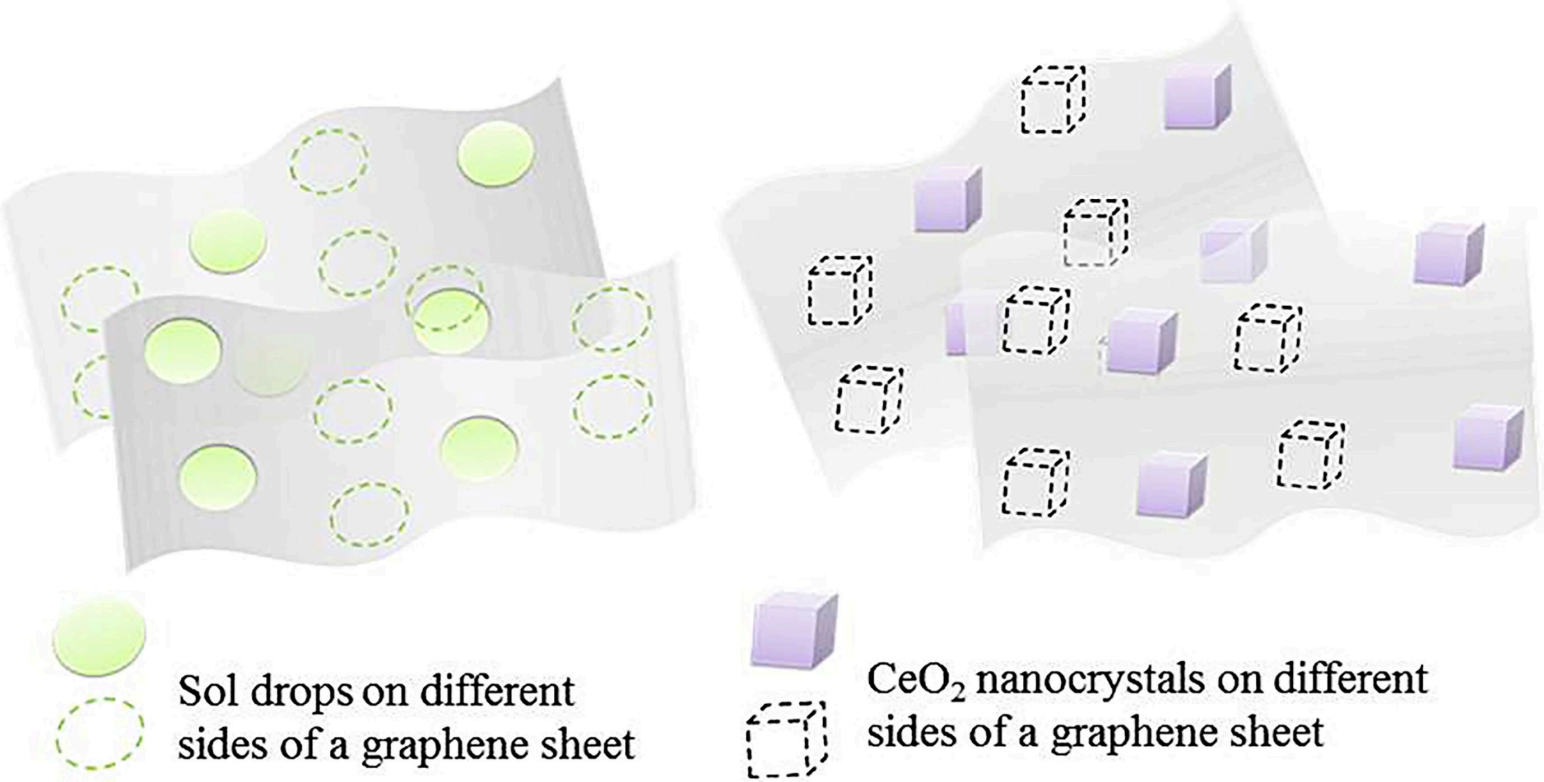
The schemes for the formation of the ‘graphene suspension—Ce-containing sol’ system (on the left) and the composite formed as a result of ceria crystallization on the graphene sheets (on the right).

Figure 13 (left) schematically shows the fixation of Ce-containing sol nanoparticles on graphene sheets, which appears to be quite regular. It was shown above by the TEM method that the sites of fixation of the sol particles are separated by almost equal sections of graphene (figure 5). Apparently, the ordered distribution of sol particles with a surface charge over the graphene sheet is due to the band gap structure of graphene [56]. This occurs as a result of the interaction between the 2*p*_*Z*_ orbitals of graphene and the positively charged outer layer of sol nanoparticles. During heat treatment, a sol → gel transition occurs on the graphene surface, and with a further increase in temperature, the metal oxide crystallization centres form in the place of the gel ‘islands’. The decomposition of the organic–inorganic complex, as a rule, is completed in the temperature range of 340−360°C. In the range of 360−500°C with isothermal holding at 500°C for 1 h, the discrete ceria crystallites are formed, and they are almost regularly distributed over the graphene sheet on both sides at the places of fixation of the initial sol nano-droplets (figure 13, right).

**Figure 14.**
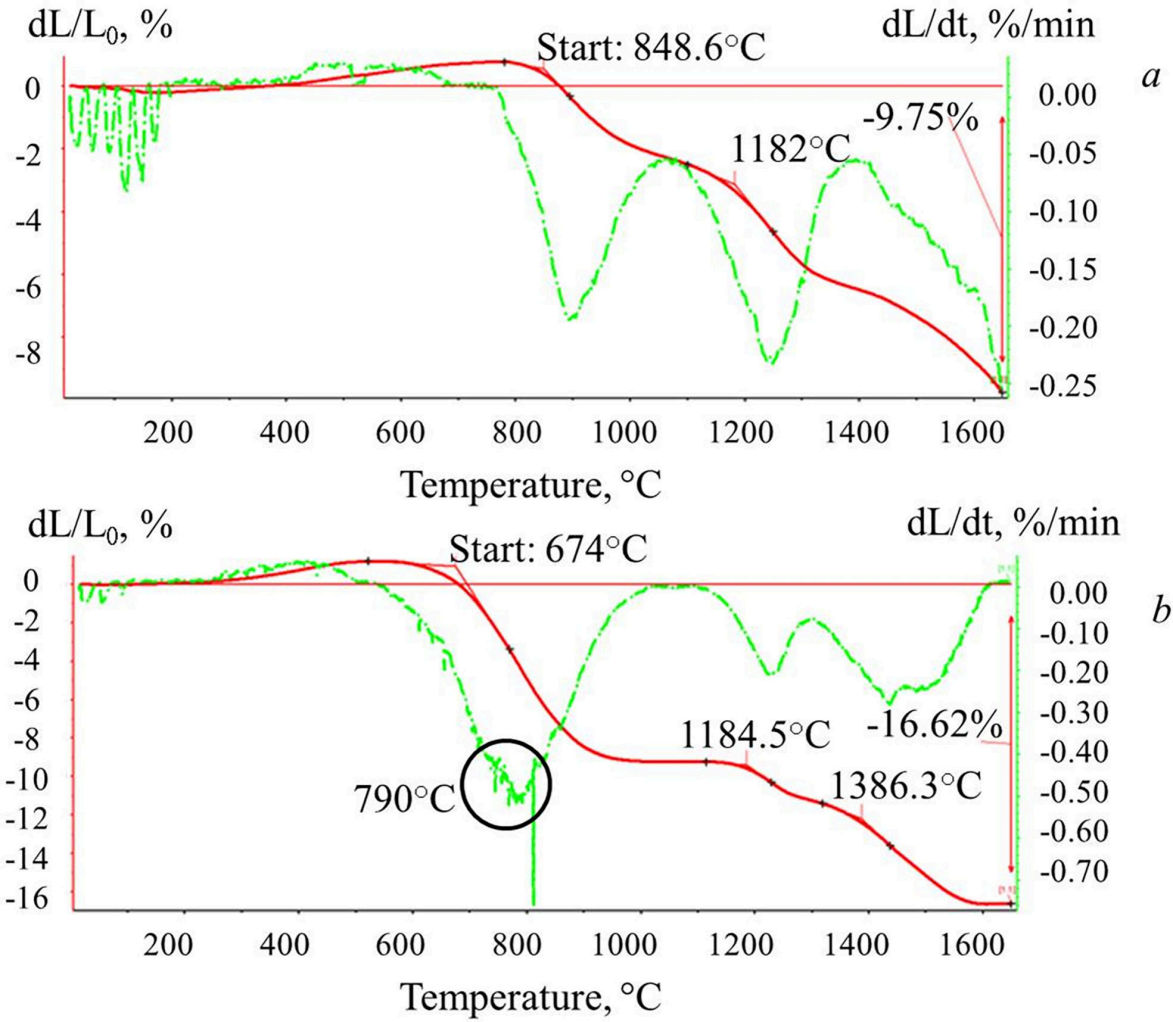
The curves of shrinkage (red) and shrinkage rate (green) for samples: pure nano-ceria (*a*) and graphene–ceria composite (*b*).

The results of a dilatometric study of powders of pure ceria and graphene–ceria composite are shown in figure 14. The shape of the shrinkage curve for pure nano-ceria (figure 14*a*) indicates that sintering proceeds in two stages, the beginning of which falls at 848 and 1182°С. The maximum shrinkage at the end of the entire cycle was at 1650°C and amounted to 9.8% of the initial linear sample dimensions. An analysis of the shrinkage and shrinkage rate curves for the graphene–ceria composite (figure 14*b*) shows that the sintering start temperature of the composite decreases by 175°C compared with the sintering start point of pure ceria. The start of composite sintering falls at 674°С, and the maximum shrinkage rate is observed at 790°С. Then the shrinkage curve reaches a plateau, after which a two-step section is also observed, as in the case of pure nano-ceria powder, with inflections at 1185 and 1386°C. In the temperature range 1600–1650°C, the curve reaches a plateau, and, as a result, the maximum shrinkage value is 16.6%. An analysis of the data obtained allows us to conclude that a graphene component contributes to sample compaction and, as a result, better sintering.

## 4. Conclusion

Thus, it was shown that the developed method for obtaining a hybrid nanostructure based on oxygen-free graphene and ceria makes it possible to achieve a uniform distribution of components in the material at the nano-level. The diffraction pattern of the composite with a graphene content equal to 0.5−0.6 wt% corresponds to a single-phase ceria with a fluorite structure in the cubic syngony. Graphene sheets are uniformly distributed in its volume at the nano-level without forming any impurity phases. It was found that the crystallization of ceria on the graphene sheets leads to an increase in the fraction of crystal lattice deformations due to the interaction of interface monolayers of crystals with the band structure of graphene. Graphene sheets also inhibit the growth of ceria crystals, the sizes of which in the hybrid are a quarter smaller than the crystallite sizes of pure nano-ceria obtained from the same sol under the same conditions. It was found that at a low content in the reaction mixture, graphene sheets are capable of destroying the system of hydrogen bonds in the aqueous part of the graphene suspension and promoting the formation of smaller ceria nanocrystallites with the dimensions of quantum dots.

For the first time, powder composites based on oxygen-free graphene and ceria were studied using the dilatometry method. It was found that the incorporation of 0.5−0.6 wt% of uniformly distributed oxygen-free graphene sheets leads to a decrease in the sintering start temperature of the composite by 175°C compared with pure nano-ceria, and the shrinkage value increases by two times, which, in turn, will contribute to better sintering of ceramics.

The resulting composite is promising in the development of new materials for small devices in demand in electronics and energy. The method of its preparation is easily adapted to the conditions of production and, in terms of environmental acceptability, surpasses the only large-scale Hummers method currently used, which requires the use of toxic reagents and a long multi-stage production cycle. This study fills a gap in the synthesis and characterization of oxygen-free graphene-based hybrids and expands the potential for graphene composites to be used as promising raw materials for small-scale devices and large-scale power plants.

## Data Availability

‘Effect of Graphene Sheets on the Physicochemical Properties of Nanocrystallite Ceria’, available on Dryad [57].
